# NKT-Like (CD3+CD56+) Cells in Chronic Myeloid Leukemia Patients Treated With Tyrosine Kinase Inhibitors

**DOI:** 10.3389/fimmu.2019.02493

**Published:** 2019-10-22

**Authors:** Jani-Sofia Almeida, Patrícia Couceiro, Nelson López-Sejas, Vera Alves, Lenka Růžičková, Raquel Tarazona, Rafael Solana, Paulo Freitas-Tavares, Manuel Santos-Rosa, Paulo Rodrigues-Santos

**Affiliations:** ^1^Faculty of Medicine (FMUC), Institute of Immunology, University of Coimbra, Coimbra, Portugal; ^2^Laboratory of Immunology and Oncology, Center for Neuroscience and Cell Biology (CNC), University of Coimbra, Coimbra, Portugal; ^3^Faculty of Medicine, Center of Investigation in Environment, Genetics and Oncobiology (CIMAGO), University of Coimbra, Coimbra, Portugal; ^4^Faculty of Medicine, Coimbra Institute for Clinical and Biomedical Research (iCBR), University of Coimbra, Coimbra, Portugal; ^5^Center for Innovation in Biomedicine and Biotechnology (CIBB), University of Coimbra, Coimbra, Portugal; ^6^Department of Immunology, IMIBIC - Reina Sofia University Hospital, University of Cordoba, Córdoba, Spain; ^7^Hematology Service, Coimbra Hospital and Universitary Centre (CHUC), Coimbra, Portugal; ^8^Immunology Unit, University of Extremadura, Cáceres, Spain

**Keywords:** chronic myeloid leukemia, tyrosine kinase inhibitors, NKT-like cells, natural cytotoxicity receptors, immune checkpoints

## Abstract

Therapy with Tyrosine Kinase Inhibitors (TKI) aiming stable deep molecular response is the gold standard to treat Chronic Myeloid Leukemia (CML). NKT-like cells (CD3^+^CD56^+^) combine characteristics of T and NK cells. The physiopathological role of these cells remains unknown although the literature refers their association with inflammation, autoimmune diseases, and cancer. Since the information regarding the role of NKT-like cells in CML is rare, we aimed at the characterization of these cells in CML patients treated with TKIs. Peripheral blood NKT-like cells from 48 CML patients and 40 healthy donors were analyzed by multiparametric flow cytometry. Functional tests consisting of co-culture with leukemic target cells (K562 cell line) were used to measure degranulation and cytokine production. Our results revealed that NKT-like cells are decreased in treated CML patients, although they present increased expression of activation markers (CD69 and HLA-DR), increased degranulation (CD107a) and impaired IFN-γ production. Significantly alterations on the expression of tumor recognition (NCRs and NKp80), and immune regulation receptors (LAG-3, TIM-3, and CD137) by NKT-like cells were observed in CML patients. Second generation TKIs increased cell activation (CD69) and decreased expression of NKp44 and NKp80 by NKT-like cells from CML patients when compared to Imatinib. CML patients that achieved deep molecular response (MR4.5) presented downregulation of NKp44 and LAG-3. Further studies are needed to clarify the role of these cells as biomarkers of therapy response and also to evaluate their value for discrimination of better candidates for sustained treatment-free remission after TKI discontinuation.

## Introduction

Chronic Myeloid Leukemia (CML) is a myeloproliferative disease that develops from a clonal expansion of hematopoietic cells in the bone marrow (BM) with consequent accumulation of immature cells in peripheral blood (1, 2). CML biology is characterized by the expression of constitutively activated tyrosine kinase proteins (3), that are encoded by the BCR-ABL oncogene (4). BCR-ABL is present in the Philadelphia chromosome (5) generated from a reciprocal *t* (9:22) translocation (6). The introduction of Imatinib and new generations of tyrosine kinase inhibitors (TKIs) represented a shift in chronic phase CML (CP-CML) treatment (7). With TKIs, an higher proportion of patients achieve long-term deep molecular responses (DMR) and the life expectancy of newly diagnosed patients gets close to age-matched normal individuals (8, 9).

Is well known that TKIs have off-target immunomodulatory effects, namely on effector and regulatory T cells, NK cells, B cells, and dendritic cells. Moreover, immune reactivation in CP-CML patients has been associated with TKI therapy (10–15). In the context of immunomodulation, the 2nd generation TKI Dasatinib is the most interesting, since it has targets that are directly implicated in immune regulation (16–19) and it is associated with large granular lymphocytosis, resulting in expansion of T CD8 and NK cell clones (20).

Natural killer T cells (usually defined as CD3^+^CD56^+^), are a poorly known, controversial and heterogeneous population that shares characteristics from both NK and T cells. The classification of NKT cells has been used to define different subpopulations of T cells expressing NK receptors, such as CD1d-restricted cells with invariant TCR (iNKT) or CD8 T cells that acquire NK receptors (NKT-like cells) (21, 22). Whereas iNKT frequency decreases, NKT-like cells increase with age in peripheral blood of healthy individuals (22). It has been shown that iNKT cells from chronic phase CML patients show functional deficiencies that are restored upon remission, although their possible contribution to disease control by TKI based therapies is unclear (23).

NKT-like cells are large granular lymphocytes, CD1d-unrestricted, possess a polyclonal TCR rearrangement, effectively kill cancer cells in a non-MHC-restricted fashion and are capable of cytokine production (21, 22, 24–26). Recent studies clearly distinguish NKT-like cells from NK, iNKT, and CD56^−^ T cells (27, 28).

Besides the lack of knowledge about NKT-like cells, some authors reported alterations in this particular population in patients with autoimmune diseases (29, 30), chronic inflammation (31), infection (32–34), and solid tumors (35, 36). There are few studies published concerning NKT-like cells in hematologic malignancies (37–39), yet in chronic lymphocytic leukemia (CLL), low numbers of NKT-like cells have been associated with disease progression (37, 38).

Considering that CML and TKI therapy induce changes in phenotype and function of immune cells (10–15), we performed extended immunophenotyping of NKT-like cells, including functional tests (degranulation and IFN-γ production), maturation, activation, and migration status markers and also detailed analysis of NKG2 family receptors, NCRs, NKp80 and immune checkpoints (ICP) expression on NKT-like cells from CML patients treated with tyrosine kinase inhibitors. We identified low numbers of NKT-like cells in peripheral blood from CP-CML patients with cytotoxic potential and differences in the repertoire of receptors. The latter was more evident for receptors linked to activation and immune regulation.

## Materials and Methods

### Patients and Healthy Donors

Peripheral blood (PB) samples were collected in heparin tubes, in average 12 h after the drug intake and analyzed within 24 h. The study group consisted of 48 PB samples from CML patients [62 ± 13 years; 21 (43.75% females)] undergoing tyrosine kinase inhibitory (TKI) therapy collected at the Hematology Service from Coimbra Hospital and Universitary Centre. PB samples from 40 healthy donors (HD) were used as control group (63 ± 12 years of age; 52.5% females). We also analyzed the impact of different generations of TKI. Detailed information regarding risk scores, therapy, and response are summarized in Supplementary File 1. All the volunteers agreed and signed informed consent to participate in the present study approved by the Ethical Committee of the Faculty of Medicine of the University of Coimbra and the Coimbra Hospital and Universitary Centre (Portugal).

### Functional Tests: Degranulation and IFN-γ Production

In aseptic conditions, effector cells (peripheral blood mononuclear cells, PBMCs) were isolated from heparinized blood samples by a standard “Ficoll-Hypaque” (Histopaque, Sigma Aldrich, UK) protocol. PBMCs were counted to adjust the concentration to 50 × 10^6^cells/mL. Target cells (K562 cell line; ATCC CCL-243, Manassas, VA, USA) were maintained in culture, viability was determined and cell concentration adjusted to 1 × 10^5^cells/mL. Into 12 × 75 mm tubes, 75 μL of effector and 100 μL of target cells were mixed in a 25:1 effector ratio, with 2 μL of anti-CD107a-PE antibody (BD Pharmingen, San Jose, CA, USA) and 1 μL of Brefeldin A (Sigma, 10 μg/mL) in a final volume of 200 μL with RPMI 1640 medium (Invitrogen, Carlsbad, CA, USA). Tubes were placed in a humidified 5% (v/v) CO_2_ incubator in the water reservoir at the bottom, for 4 h at 37°C. At the end of incubation cells were washed and resuspended in 100 μL of 1 × phosphate-buffered saline (PBS; Invitrogen, Carlsbad, CA, USA) and the extracellular antibodies were added, anti-CD56-PerCP-Cy5.5 (BD Pharmingen, San Jose, CA) and anti-CD3-V500 (BD Horizon, San Jose, CA, USA). After 15 min of incubation in the dark at room temperature (RT), suspensions were treated with Fix and Perm A solution (Invitrogen, Carlsbad, CA, USA) for 15 min, in the dark at RT. Cells were centrifuged at 453 g for 5 min and the supernatant discarded. Next, cells were incubated with Fix and Perm B solution (Invitrogen, Carlsbad, CA, USA) and the intracellular antibodies anti-IFN-γ-V450 (BD Horizon, San Jose, CA, USA) and anti-Granzyme-B-FITC (BD Pharmingen, San Jose, CA, USA) for 20 min, in the dark at RT. After centrifugation at 453 g for 5 min and supernatant discarded, cells were resuspended in 1 × PBS and acquired in FACS Canto II with DIVA software (Becton Dickinson, San Jose, CA, USA). For each sample, in the same conditions, effector cells were incubated without target cells to detect spontaneous degranulation and cytokine production.

### Immunophenotyping

White blood cells from fresh peripheral blood samples were counted in the hematological counter (A^c^T diff, Beckman Coulter, Brea, CA, USA). 100 uL or up to 1 × 10^6^ cells were added to 12 × 75 mm tubes and stained with the extracellular antibodies for 15 min, in the dark at RT. After incubation red blood cells were lysed with BD Lysing Solution (Becton Dickinson, San Jose, CA, USA), for 10 min. Cell suspensions were centrifuged and washed one time with 1 × PBS in same conditions. In the end, cell suspensions were acquired in FACS Canto II with DIVA software (Becton Dickinson, San Jose, CA, USA). The antibodies used for cellular staining are described in Supplementary File 2.

### Data and Statistical Analysis

Flow cytometry data was analyzed with FlowJo v.10.7 (Tree Star, Ashland, OR, USA). The gating strategy used for the functional assays and immunophenotyping is similar. Lymphocytes were discriminated based on morphologic parameters SSC-A and FSC-A and doublets excluded using FSC-H and FSC-A. Gated on lymphocytes, NKT-like cells were identified by the expression of both CD3 and CD56 receptors. Within the NKT-like population, it was determined the frequency of positive cells and the median fluorescence intensity (MFI) of CD11b, CD16, CD27, CD56, CD57, CD62L, CD69, CD107a, CD137, CD137L, CRACC, HLA-DR, LAG-3, NKG2A, NKG2C, NKG2D, NKp30, NKp44, NKp46, NKp80, PD-1, TIM-3, and the production of Granzyme-B and IFN-γ. The cut-off for extracellular receptors or intracellular proteins was defined using isotype controls specific for each marker (described in Supplementary File 2). The statistical analysis was performed with GraphPad Prism version 8.0.1 for Windows (GraphPad Software, San Diego, CA, USA) and data is presented as mean ± standard deviation. Paired *t*-test and Mann Whitney *U*-test was used to compare means between two groups. One-way ANOVA followed by Dunn's multiple comparisons test was used to compare more than two groups and Spearman correlation to evaluate the association between two parameters. ClustVis was accessed online (https://biit.cs.ut.ee/clustvis) for visualizing clustering of multivariate data using Principal Component Analysis and heatmaps (40). Original values were ln(x+1)-transformed. Unit variance scaling was applied to rows; singular value decomposition (SVD) with imputation was used to calculate principal components. X and Y axis show principal component 1 and principal component 2 that explain the indicated percentages of the total variance. Prediction ellipses are such that with probability 0.95, a new observation from the same group will fall inside the ellipse. Clustering distances were obtained using Pearson correlation subtracted from 1. Ward linkage method was calculated using sum of squared differences from points to centroids as the distance.

## Results

No statistically significant differences were observed for CML patients concerning absolute and relative frequencies of NKT-like cells according to sex, age, sample time after diagnosis (Supplementary File 3) and clinical risk scores Sokal (41), EURO (42), EUTOS (43), and ELTS (44) (Supplementary File 4).

**Figure 1:**
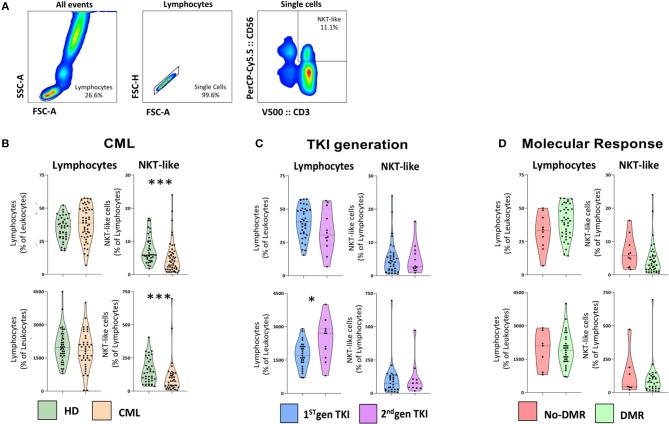
NKT-like cells are decreased in treated CML patients. Heparinized fresh whole blood samples were stained with extracellular antibodies and analyzed by multiparametric flow cytometry. **(A)** Gating strategy to identify NKT-like population. From left to right, gate on lymphocytes, followed by single cells selection and NKT-like identification (CD3+CD56+). **(B)** CML vs. HD—Relative and absolute frequency of lymphocytes and NKT-like cells in CML patients (*n* = 48) and HD (*n* = 40). **(C)** TKI generation—Relative and absolute frequency of lymphocytes and NKT-like cells in 1st (*n* = 36) and 2nd TKI generation CML patients (*n* = 12). **(D)** Molecular Response—Relative and absolute frequency of lymphocytes and NKT-like cells in CML patients that achieved deep molecular response (DMR; *n* = 38) and with no DMR (No-DMR; *n* = 10). Mann Whitney *U*-test was used for statistical analysis and the charts represent the mean ± standard deviation for HD (*n* = 40) and CML (*n* = 48) group. HD, Healthy donors; CML, Chronic Myeloid Leukemia patients; TKI, Tyrosine Kinase Inhibitor; 1st gen. TKI, 1st generation TKI CML patients; 2nd gen. TKI, 2nd generation TKI CML patients; DMR, deep molecular response; no-DMR, without deep molecular response; *p*-value <0.05*, <0.01**, <0.001***, or <0.0001****.

Similarly, no statistically significant differences were observed between individual TKIs and after analysis of CML patients according to therapy response (Supplementary File 5). Nevertheless, we further analyzed NKT-like cells parameters considering TKI generation and deep molecular response (DMR). The gating strategy to identify NKT-like cells is shown in Figure 1A.

### NKT-Like Cells Are Decreased in CML and Present a Different Receptor Repertoire According to TKI Generation and Molecular Response

We analyzed the relative and absolute frequencies of lymphocyte and NKT-like populations in peripheral blood from CML patients and healthy donors (HD) (Figure 1B). Our results revealed no significant differences in the relative and absolute frequencies of lymphocytes in treated patients when compared to healthy donors. The relative and absolute frequency of NKT-like cells from treated CML patients [(7.5 ± 4.0 vs. 5.1 ± 4.9; *p* = 0.0004) (150 ± 91 vs. 103±129; *p* = 0.0007), respectively] are diminished compared to healthy donors, with strong statistical value (Figure 1B). However, patients treated with 2nd generation of TKIs revealed a significant increase in the absolute count of lymphocytes when compared to those treated with 1st generation TKIs (2,391 ± 917 vs. 1,737 ± 587; *p* = 0.0391) (Figure 1C). No significant difference was observed in NKT-like cells for CML patients treated with 2nd generation TKIs (Figure 1C). Similarly, no correlation was observed in lymphocytes neither in NKT-like cells for CML patients that achieved deep molecular response to therapy (Figure 1D).

**Figure 2:**
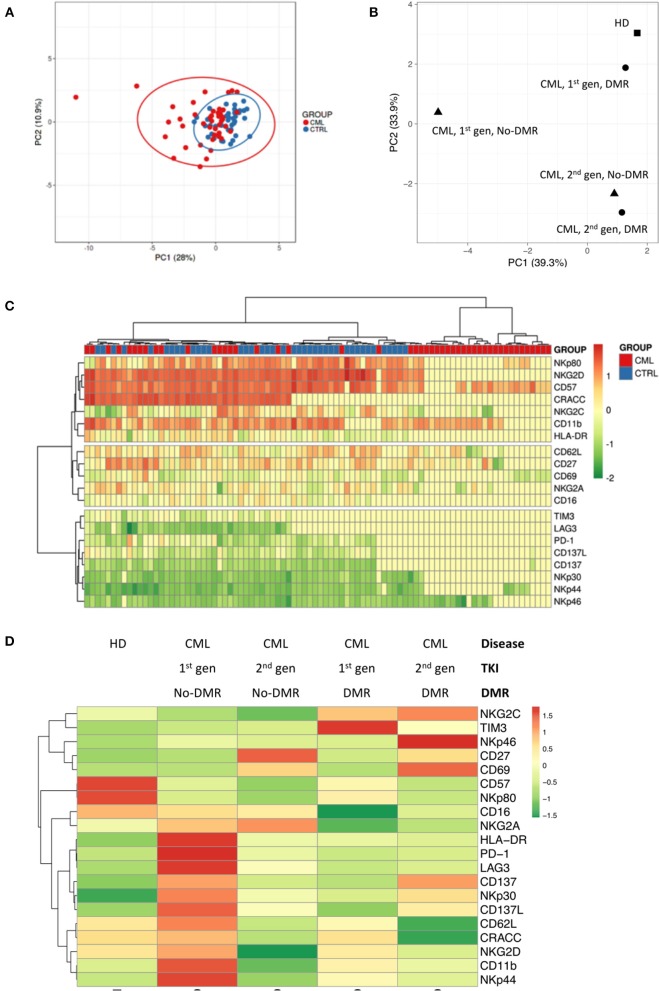
Different NKT-like cell receptor repertoire patterns in CML patients according to TKI generation and molecular response in CML. Heparinized fresh whole blood samples were stained with extracellular antibodies and analyzed by multiparametric flow cytometry. **(A)** Principal component analysis for the frequency of NKT-like cells expressing CD11b, CD27, CD57, CD62L, CD16, CRACC, CD69, HLA-DR, NKG2A, NKG2C, NKG2D, NKp30, NKp44, NKp46, NKp80, PD-1, TIM-3, LAG-3, CD137, and CD137L in CML patients (*n* = 48) and HD (*n* = 40). **(B)** Principal component analysis for the density (MFI) of CD11b, CD27, CD57, CD62L, CD16, CRACC, CD69, HLA-DR, NKG2A, NKG2C, NKG2D, NKp30, NKp44, NKp46, NKp80, PD-1, TIM-3, LAG-3, CD137, and CD137L expressed by NKT-like cells in HD (*n* = 40) and CML patients (*n* = 48) according to TKI generation [1st (*n* = 36) or 2nd (*n* = 12)] and molecular response [DMR (*n* = 38) or No-DMR (*n* = 10)]. **(C)** Heatmap for the density of CD11b, CD27, CD57, CD62L, CD16, CRACC, CD69, HLA-DR, NKG2A, NKG2C, NKG2D, NKp30, NKp44, NKp46, NKp80, PD-1, TIM-3, LAG-3, CD137, and CD137L receptors expressed by NKT-like cells in HD and CML patients. **(D)** Heatmap for the average density of CD11b, CD27, CD57, CD62L, CD16, CRACC, CD69, HLA-DR, NKG2A, NKG2C, NKG2D, NKp30, NKp44, NKp46, NKp80, PD-1, TIM-3, LAG-3, CD137, and CD137L receptors expressed by NKT-like cells in HD and CML patients according to TKI generation and molecular response. HD, Healthy donors; CML, Chronic Myeloid Leukemia patients; TKI, Tyrosine Kinase Inhibitor; 1st gen. TKI, 1st generation TKI CML patients; 2nd gen. TKI, 2nd generation TKI CML patients; DMR, deep molecular response; no-DMR, without deep molecular response.

Principal Component Analysis using relative frequency of NKT-like cell subsets, according to the markers used in this study (Figure 2A) demonstrated that CML patients cluster with HD, although more dispersed than those control samples. Similarly, combined analysis of TKI generation and molecular response (Figure 2B), cluster 1st generation TKI CML patients that achieved DMR closer to HD. The figure also shows vicinity of CML patients treated with 2nd generation TKIs, independently of molecular response.

Cluster analysis revealed by heatmaps identified different patterns for the density of receptors expressed by NKT-like cells (Figure 2C) when comparing CML patients with HD. Combined analysis of TKI generation and molecular response (Figure 2D) revealed different patterns of NKT-like cell expression in the analyzed groups.

### Increased Degranulation and Decreased IFN-γ Production by NKT-Like Cells in Treated CML Patients

**Figure 3:**
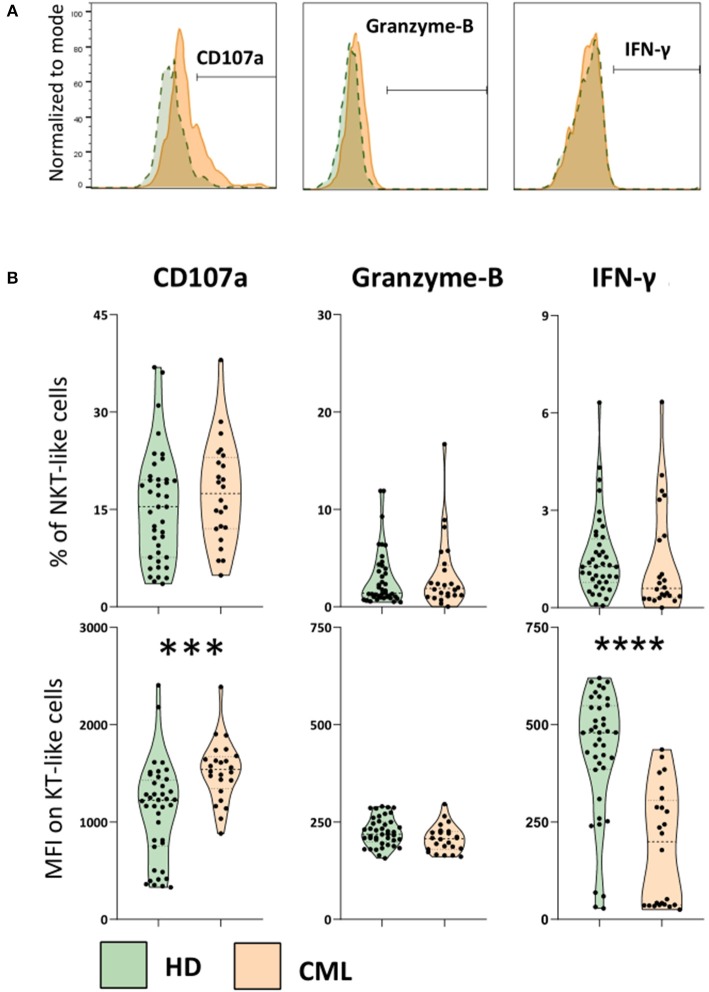
Increased degranulation and decreased IFN-γ production by NKT-like cells in treated CML patients. PBMCs isolated from heparinized fresh whole blood were incubated with and without the K562 cell line (effector:target ratio 25:1), in the presence of Brefeldin A, for 4 h in CO_2_ incubator, at 37°C. At the end of incubation cells were stained for cell membrane receptors and intracellular targets to be analyzed by multiparametric flow cytometry. **(A)** Representative histograms of the cut-off for positive NKT-like cells for each parameter. From left to right: CD107a expression, Granzyme-B and IFN-γ intracellular production from CML patients (orange) and HD group (green). **(B)** Top: Frequency of CD107a NKT-like cells, intracellular accumulation of Granzyme-B and IFN-γ intracellular production against K562 cell line. Bottom: Corresponding MFI of CD107a, Granzyme-B and IFN-γ parameters. Graphs represent data from HD (*n* = 40) and CML (*n* = 24) samples as mean ± standard deviation. Paired *t*-test was used to compare between groups. HD, Healthy donors; CML, treated CML patients; MFI, Median Intensity Fluorescence; *p*-value <0.05*, <0.01**, <0.001***, or <0.0001****.

In order to evaluate NKT-like cytotoxicity and cytokine production, we performed an indirect cytotoxic assay (CD107a, degranulation test) after 4 h co-culture with K562 cell line (Figure 3). Adding Brefeldin A, we also measured Granzyme-B and IFN-γ accumulated at the Golgi complex/endoplasmic reticulum of NKT-like cells in the same conditions. Although our results do not revealed significant differences in the percentage of NKT-like degranulating cells, we observed a significant increase of CD107a expression by NKT-like cells when comparing treated patients with HD (MFI: 1,109 ± 495 vs. 1,525 ± 315; *p* = 0.0002) (Figure 3B). Contrarily, IFN-γ produced by NKT-like cells from treated patients were decreased compared to healthy donors (MFI: 427 ± 164 vs. 118 ± 149; *p* < 0.0001) (Figure 3B). Granzyme-B produced by NKT-like cells *in vitro* was not affected in CML treated patients (Figure 3B). No effect of 2nd generation TKIs and deep molecular response was observed for CD107a, Granzyme B, and IFN-γ (data not shown).

### Maturation and Migration Markers Expressed by NKT-Like Cells in CML

We analyzed the expression of surface markers on NKT-like cells, indicative of NK and T cells maturation (CD11b, CD27, and CD57) and migration (CD62L). CML patients under treatment with 2nd generation TKIs presented a significant increase in the density of CD62L on NKT-like cells when compared to CML patients treated with 1st generation TKI (MFI: 113 ± 42 vs. 170 ± 98; *p* = 0.0322) (Supplementary File 6). No other significant differences for relative frequencies or surface receptor densities were identified in treated CML group when compared with healthy individuals (data not shown).

### NKT-Like Cells Express Activation Markers in Treated CML Patients

**Figure 4:**
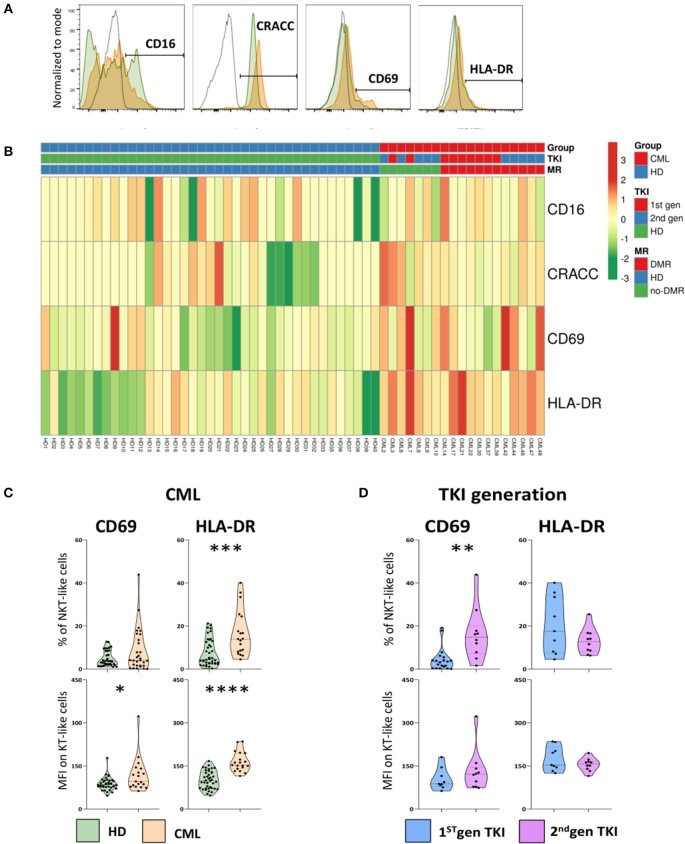
Increased activation of NKT-like cells in treated CML patients. Heparinized fresh whole blood samples were stained with extracellular antibodies and analyzed by multiparametric flow cytometry. **(A)** Representative histograms relative to CD16, CRACC, CD69, and HLA-DR expression by NKT-like cells. The dashed black lines represent the isotype control, the fulfilled green lines represent the HD group and the fulfilled red lines represent the CML group. **(B)** Heatmaps for the frequency of NKT-like cells expressing CD16, CRACC, CD69, and HLA-DR in HD (*n* = 40) and CML patients (*n* = 48) according to TKI generation [1st (*n* = 36) or 2nd (*n* = 12)] and molecular response [DMR (*n* = 38) or No-DMR (*n* = 10)]. **(C)** Relative frequencies and MFI of CD69 and HLA-DR in the NKT-like population from CML patients (*n* = 19) and HD (*n* = 38 and *n* = 40, respectively). **(D)** Relative frequencies and MFI of CD69 and HLA-DR in the NKT-like population according to 1st generation TKI (*n* = 20 and *n* = 9, respectively) and 2nd generation TKI (*n* = 10). Mann Whitney *U*-test was used for statistical analysis and the charts represent the mean ± standard deviation. HD, Healthy donors; CML, treated CML patients; TKI, Tyrosine Kinase Inhibitor; 1st gen. TKI, 1st generation TKI CML patients; 2nd gen. TKI, 2nd generation TKI CML patients; MFI, Median Intensity Fluorescence; *p*-value <0.05*, <0.01**, <0.001***, or <0.0001****.

The expression of surface receptors linked to activation of NK and T cells were also analyzed on NKT-like cells (Figure 4). The gating strategy to identify activation markers expressed by NKT-like cells and heatmap showing their relative frequency distribution according to disease, TKI generation and molecular response are shown in Figures 4A,B, respectively.

When compared to HD, treated CML patients presented a significant increase in the relative frequency of HLA-DR NKT-like cells (Percentage: 7.6 ± 6.2 vs. 16.5 ± 10.6; *p* = 0.0005) and surface density of HLA-DR (MFI: 101 ± 31 vs. 164 ± 34; *p* < 0.0001) (Figure 4C).

Additionally, CD69 density on the surface of NKT-like cells from treated CML patients was found significantly increased (MFI: 87.9 ± 22.4 vs. 118.0 ± 59.4; *p* = 0.00467) when compared to HD (Figures 4B,C). CML patients treated with 2nd generation TKIs, revealed a significant increase of CD69^+^ NKT-like cells (Percentage: 16.2 ± 12.2 vs. 4.4 ± 5.3; *p* = 0.0022] (Figure 4D).

No alterations were observed for the expression of CD16 and CD2-like receptor-activating cytotoxic cell (CRACC) receptor, a co-stimulatory molecule, on NKT-like cells (Figure 4B).

### NKG2 Receptor Family on NKT-Like Cells in CML Patients

Concerning the NKG2 family, characteristic from NK cells, we evaluated the expression of the inhibitory receptor NKG2A and the activating receptors NKG2C and NKG2D on NKT-like cells from CML patients. No differences were found either in the frequency of NKT-like cells expressing these receptors or in the receptor surface density between the groups analyzed (data not shown).

### NCRs and NKp80 Are Significantly Altered on NKT-Like Cells in CML Patients

**Figure 5:**
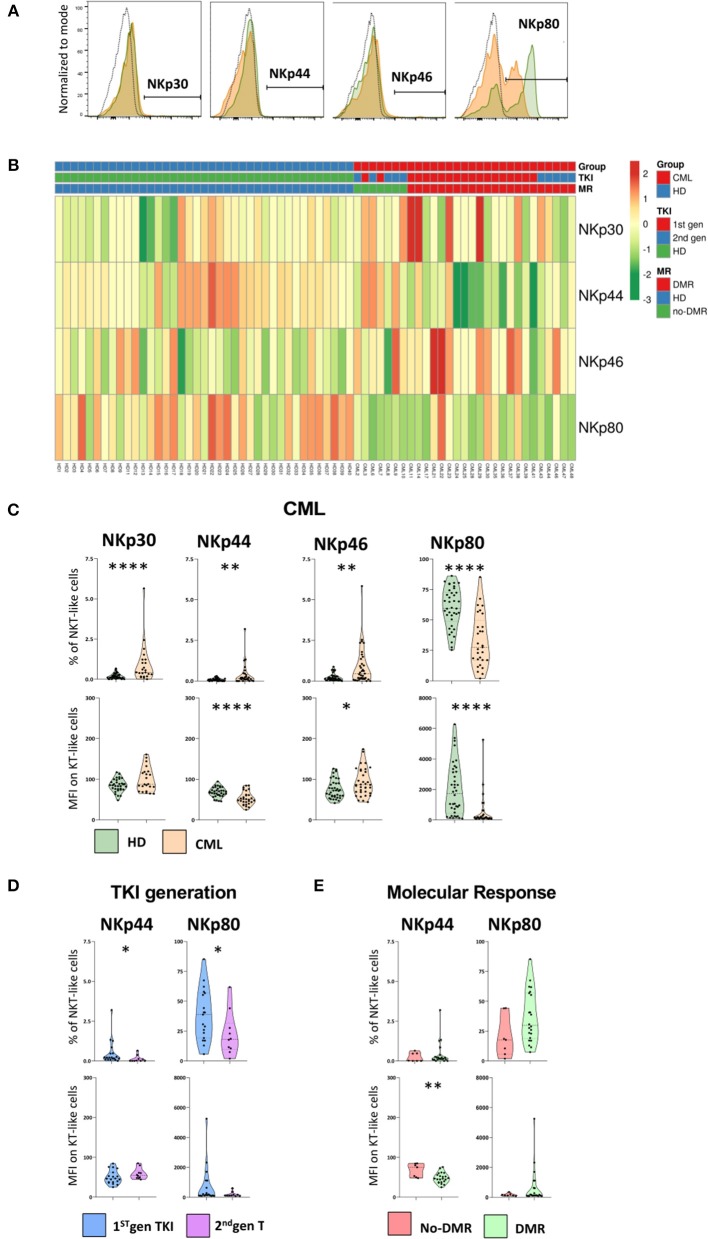
NCRs and NKp80 are significantly altered on NKT-like cells in CML patients. Heparinized fresh whole blood samples were stained with extracellular antibodies and analyzed by multiparametric flow cytometry. **(A)** Representative histograms relative to the cut-off for positive NKT-like cells in terms of, from left to right, NKp30, NKp44, NKp46, and NKp80. The dashed black lines represent the isotype control, the fulfilled green lines represent the HD group and the fulfilled red lines represent the CML group. **(B)** Heatmaps for the frequency of NKT-like cells expressing NKp30, NKp44, NKp46, and NKp80 in HD (*n* = 40) and CML patients (*n* = 48) according to TKI generation [1st (*n* = 36) or 2nd (*n* = 12)] and molecular response [DMR (*n* = 38) or No-DMR (*n* = 10)]. **(C)** NKp30, NKp44, NKp46 and NKp80 relative frequencies and MFI of NKT-like population from CML patients (*n* = 23, *n* = 29, *n* = 36 and *n* = 29, respectively) and HD (*n* = 39, *n* = 39, *n* = 39 and *n* = 38, respectively). **(D)** Relative frequencies and MFI of NKp44 and NKp80 in the NKT-like population according to 1st gen. TKI (*n* = 19) and 2nd gen. TKI (*n* = 10). **(E)** Relative frequencies and MFI of NKp44 and NKp80 of NKT-like cells from patients without deep molecular response (*n* = 9) or with deep molecular response (*n* = 25). Mann Whitney *U*-test was used for statistical analysis and the charts represent the mean ± standard deviation. HD, Healthy donors; CML, treated CML patients; TKI, Tyrosine Kinase Inhibitor; 1st gen. TKI, 1st generation TKI CML patients; 2nd gen. TKI, 2nd generation TKI CML patients; DMR, deep molecular response; no-DMR, without deep molecular response; MFI, Median Intensity Fluorescence; *p*-value <0.05*, <0.01**, <0.001***, or <0.0001****.

The gating strategy to identify NCR^+^ and NKp80^+^ NKT-like cells and heatmap showing their relative frequency distribution according to disease, TKI generation and molecular response are shown in Figures 5A,B, respectively.

In spite of the very low or absent expression of natural cytotoxic receptors (NCRs: NKp30, NKp44, and NKp46) on NKT-like cells, we found significant alterations in CML patients (Figure 5C). Comparing to HD, treated CML patients exhibited an increase of NKT-like cells expressing NKp30 (Percentage: 0.2 ± 0.1 vs. 0.9 ± 1.2; *p* < 0.0001), NKp44 (Percentage: 0.1 ± 0.1 vs. 0.4 ± 0.6; *p* = 0.007), and NKp46 (Percentage: 0.2 ± 0.2 vs. 0.9 ± 1.2; *p* = 0.002) receptors (Figure 5C). A significant decrease in MFI of NKp44 (MFI: 68.2 ± 11.7 vs. 52.9 ± 16.1; *p* = 0.00022) and increase of NKp46 (MFI: 92.3 ± 33.3 vs. 75.63 ± 23.21; *p* = 0.0233) surface density was observed, comparatively to healthy donors (Figure 5C).

Moreover, the expression of co-stimulatory molecule NKp80 on NKT-like cells of treated patients had a significant reduction in the frequency of NKp80 NKT-like cells (Percentage: 59.9 ± 16.3 vs. 32.9 ± 21.4; *p* < 0.0001) as well as in the density per cell (MFI: 1,985 ± 1,698 vs. 550 ± 1,053; *p* < 0.0001) (Figure 5C).

Second generation TKIs decreased significantly NKp44 (Percentage: 0.1 ± 0.2 vs. 0.5 ± 0.8; *p* = 0.0137) and NKp80 NKT-like cells (Percentage: 22.4 ± 18.2 vs. 38.4 ± 21.3; *p* = 0.0459) (Figure 5D).

CML patients achieving deep molecular response showed (Figure 5E) a significant reduction of the density of NKp44 at the surface of NKT-like cells (MFI: 48.1 ± 12.9 vs. 67.9 ± 16.8; *p* = 0.0101).

### Immune Checkpoints Expressed by NKT-Like Cells in CML Patients

**Figure 6:**
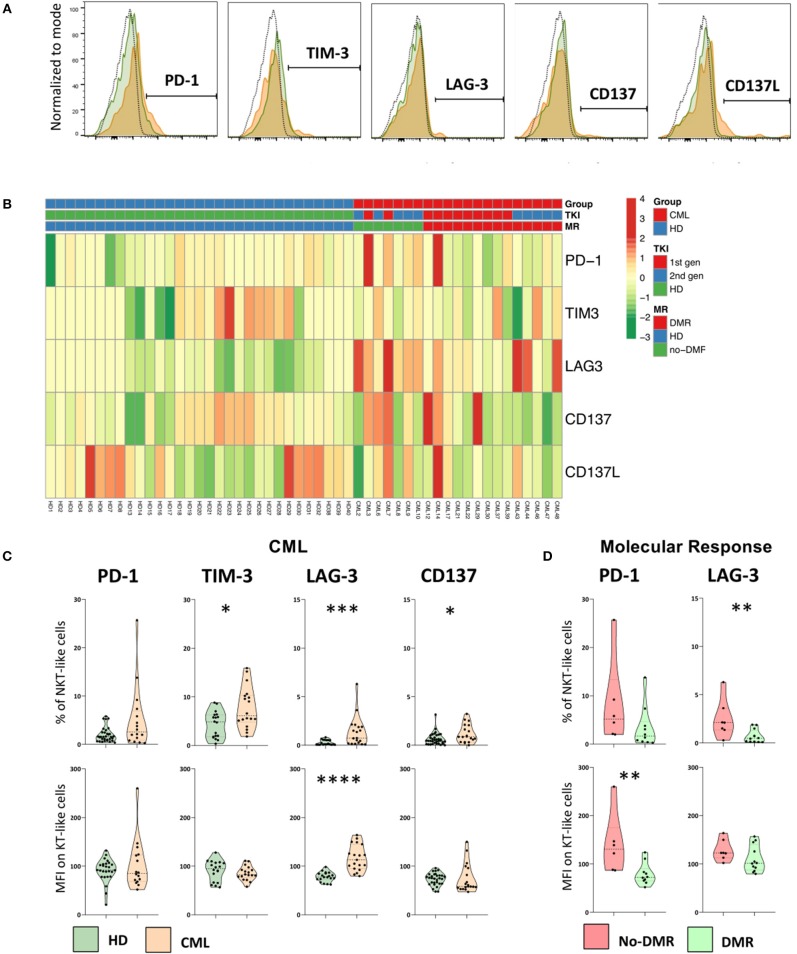
Immune checkpoints are expressed by NKT-like cells in CML patients and are decreased in patients achieving deep molecular response. Heparinized fresh whole blood samples were stained with extracellular antibodies and analyzed by multiparametric flow cytometry. **(A)** Representative histograms relative to the cut-off for positive NKT-like cells in terms of, from left to right, PD-1, TIM-3, LAG-3, CD137, and CD137L. The dashed black lines represent the isotype control, the fulfilled green lines represent the HD group and the fulfilled red lines represent the CML group. **(B)** Heatmaps for the frequency of NKT-like cells expressing PD-1, TIM-3, LAG-3, CD137, and CD137L in HD (*n* = 40) and CML patients (*n* = 48) according to TKI generation [1st (*n* = 36) or 2nd (*n* = 12)] and molecular response [DMR (*n* = 38) or No-DMR (*n* = 10)]. **(C)** PD-1, TIM-3, LAG-3 and CD137 relative frequencies and MFI of NKT-like population from CML patients (*n* = 16, *n* = 18, *n* = 19 and *n* = 19, respectively) and HD (*n* = 32, *n* = 18, *n* = 19 and *n* = 35, respectively). **(D)** Relative frequencies and MFI of PD-1 and LAG-3 of NKT-like cells from patients without deep molecular response (*n* = 6 and *n* = 7, respectively) or with deep molecular response (*n* = 10 and *n* = 12, respectively). Mann Whitney *U*-test was used for statistical analysis and the charts represent the mean and standard deviation. HD, Healthy donors; CML, treated CML patients; DMR, deep molecular response; no-DMR, without deep molecular response; MFI, Median Intensity Fluorescence; *p*-value <0.05*, <0.01**, <0.001*** or <0.0001****.

The gating strategy to identify immune checkpoints expressed by NKT-like cells and heatmap showing their relative frequency distribution according to disease, TKI generation and molecular response are shown in Figures 6A,B, respectively.

We found increased expression of TIM-3 (Percentage: 4.3 ± 2.7 vs. 7.7 ± 4.2; *p* = 0.0169), LAG-3 (Percentage: 0.3 ± 0.3 vs. 1.4 ± 1.5; *p* = 0.0013), and CD137 (Percentage: 0.6 ± 0.6 vs. 1.2 ± 0.9; *p* = 0.0326) in the NKT-like population from treated CML patients, when compared to HD group (Figure 6C). In addition, it was also observed a higher density of LAG-3 (MFI: 78 ± 10 vs. 117 ± 26; *p* < 0.0001) (Figure 6C).

In CML patients with deep molecular response, it was observed a decrease in the frequency of LAG-3 NKT-like cells (Percentage: 0.7 ± 0.7 vs. 2.5 ± 2.0; *p* = 0.0127) and lower density of PD-1 on the surface of these cells (MFI: 79 ± 22 vs. 140 ± 63; *p* = 0.0047) (Figure 6D) when compared to patients without deep molecular response. In our study no differences were observed regarding CD137L expression by NKT-like cells in the groups of study (data not shown).

## Discussion

In CML, the TKI-dependent mechanisms underlying immune regulation are not clear. However, it is widely recognized that TKIs have immunomodulatory effects that influence response to therapy and also according to drug generation (10–14). Several authors reported the immune restoration of PB lymphocytes levels in treated CML patients and also the particular effect of 2nd generation TKI (14, 15, 39). Our results corroborate the preceding studies. We observed normal lymphocyte counts in TKI treated CML patients that were more evident for patients undergoing 2nd generation TKIs.

To our knowledge, there is only one study that analyzed NKT-like cells as a whole population in CML and described no differences in PB of TKI treated patients, with exception of those under Dasatinib (39). Our results revealed a decrease in relative and absolute frequency of NKT-like cells in PB of treated CML patients without discrimination between first or second generation TKI. This discrepancy can be explained by the significant difference in the sample time after diagnosis between the two CML studies.

In our study, NKT-like cells from treated CML patients exposed to the leukemic cell line K562 upregulated CD107a expression and decreased IFN-γ production. Lack of other studies for comparisons allows us to speculate that NKT-like cells had increased cytotoxicity as a major TKI-dependent anti-leukemic effect. No obvious explanation for the significant downregulation of IFN-γ recommends further studies in order to clarify these results.

Concerning CD62L, a molecule correlated with migration, we observed that patients treated with 2nd generation TKIs had lower density of CD62L on NKT-like cells. This is in line with CML studies on T cells that demonstrated downregulation of CD62L after TKI therapy (45). In particular, it has been reported that dasatinib-treated patients had less CD62L-expressing CD4+ and CD8+ T cells (39, 46) whereas in nilotinib-treated patients, the expression levels of CD62L were similar as in imatinib-treated patients.

CRACC receptor could act as activator or inhibitory trigger in immune cells depending on the microenvironment (47). Our results do not suggest a clear role for CRACC NKT-like cells in treated CML patients. We assumed CD69 and HLA-DR expression by NKT-like cells as the classical early/late activation markers on T cells, respectively. Thus, we demonstrated that treated patients have more HLA-DR+ NKT-like cells and more HLA-DR per cell and revealed a higher density of CD69. The expression of these activating markers was in accordance with our results that showed higher degranulation by NKT-like cells from treated CML patients.

Despite the very low expression of NCRs, we found higher frequencies of NKp30, NKp44, and NKp46 NKT-like cells and increased surface density of NKp46 in our group of treated CML patients. The previous data suggest a more activated and anti-tumor profile of NKT-like cells from treated CML patients. On the other hand, we found that these cells express less NKp44. This receptor was both diminished by 2nd generation TKIs and in CML patients achieving deep molecular response. Additionally, the most evident finding regarding NK receptors was the significant decrease in both frequency of NKp80 NKT-like cells and density of receptor expression in TKI treated CML patients. In our study, downregulation of NKp80 was also associated to 2nd generation TKIs. It has been speculated that leukemic cells can self-downregulate the expression of NKp80 ligand preventing NKp80-recognition (48).

The expression of immune checkpoints (ICP) on lymphocytes is associated with immune regulation and, in line with our results, some authors reported the upregulation of ICP associated with immune suppression on PB T cells from CML patients or patients with other hematologic malignancies (49–53). To our knowledge, there are no studies analyzing the expression of ICP on NKT-like cells in CML. In spite of the very low or absent expression of ICP on NKT-like cells, we observed an increased fraction of NKT-like cells expressing TIM-3 and/or LAG-3 in treated CML patients and decreased expression of LAG-3 associated to deep molecular response.

**Figure 7:**
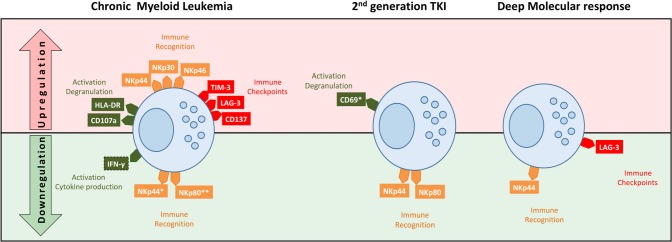
Schematic representation of NKT-like cell profiles in treated CML according to TKI generation and deep molecular response. NKT-like cells from CML patients had different patterns of receptor expression associated with function/activation (CD107a, IFN-γ, HLA-DR, and CD69), tumor recognition (NKp30, NKp44, NKp46, and NKp80) and immune regulation (TIM-3, LAG-3, CD137). Second generation TKIs were associated with remarkably activation status (CD69) and downregulation of NKp44 and NKp80. Patients in deep molecular response were associated with downregulation of NKp44 and LAG-3. Top (in red) of the diagram represents upregulation of receptors and the bottom (in green) represent the downregulation of receptors by NKT-like cells.

In summary, we studied TKI treated CML patients and found decreased frequency of NKT-like cells, higher cytotoxicity, and different patterns of membrane receptors (Figure 7). Major differences in receptor expression were associated with activation (HLA-DR, CD69), tumor recognition (NCRs and NKp80), and immune regulation (TIM-3 and LAG-3). Second generation TKIs were associated with remarkably activation status (CD69) and downregulation of NKp44 and NKp80. Deep molecular response correlates with downregulation of LAG-3. Those could represent potential markers for sustained treatment-free remission (TFR) after discontinuation.

Further studies are needed to clarify the importance of the NKT-like mechanisms cells involved in CML. Unmet needs include the analysis of the interactions between NKT-like cells and leukemic cells, the effect of TKIs on specific NKT-like cells pathways affecting function and phenotype, and the role of NKT-like cells on TKI discontinuation.

## Data Availability Statement

All datasets generated for this study are included in the manuscript/Supplementary Files.

## Ethics Statement

All the volunteers agreed and signed informed consent to participate in the present study approved by the Ethical Committee of the Faculty of Medicine of the University of Coimbra and the Coimbra Hospital and University Centre (Portugal).

## Author Contributions

J-SA, MS-R, and PR-S: research study design. J-SA, PC, VA, and PR-S: experiments conduction and data acquisition. LR and PF-T: clinical data and patient management. J-SA, VA, NL-S, RT, RS, MS-R, and PR-S: data analysis. PR-S and MS-R: reagents providing. J-SA, RT, RS, MS-R, and PR-S: manuscript writing. All authors approved the final version of the manuscript.

### Conflict of Interest

The authors declare that the research was conducted in the absence of any commercial or financial relationships that could be construed as a potential conflict of interest.
