# Supplementary material for: NKT-Like (CD3+CD56+) Cells in Chronic Myeloid Leukemia Patients Treated With Tyrosine Kinase Inhibitors
Source: Front Immunol. 2019 Oct 22;10:2493. doi: 10.3389/fimmu.2019.02493 (PMC6817724; doi:10.3389/fimmu.2019.02493)
Supplement: Supplementary file 1 [file Data_Sheet_1.PDF]

S1. Characteristics of CML patients and healthy donors included in this study.

Characteristic, unit	*Median (SD) or n (%)*	
	**CML**	**CTRL**
Samples, n	48	40
Age, years	62 ± 13	63 ± 12
Sex (proportion female)	21 (43.75%)	21 (52.5%)
**Risk scores**		
Sokal[41], n (%)		
Low	*18 (37.50%)*	
Intermediate	*17 (35.42%)*	
High	*7 (14.58%)*	
NA	*6 (12.50%)*	
EURO[42], n (%)		
Low	*19 (39.58%)*	
Intermediate	*22 (45.83%)*	
High	*1 (2.08%)*	
NA	*6 (12.50%)*	
EUTOS[43], n (%)		
Low	*38 (79.17%)*	
High	*5 (10.42%)*	
NA	*5 (10.42%)*	
ELTS[44], n (%)		
Low	*28 (58.33%)*	
Intermediate	*12 (25.00%)*	
High	*3 (6.25%)*	
NA	*5 (10.42%)*	
**Therapy**		
1st generation TKI		
Imatinib	*36 (75%)*	
2nd generation TKI	12 (25%)	
Dasatinib	*5 (10.41%)*	
Bosutinib	*5 (10.41%)*	
Nilotinib	*2 (4.17%)*	
**Response**		
Deep Molecular Response (DMR)		
MR4.5	38 (79.17%)	
No-Deep Molecular Response (no-DMR)	10 (20.83%)	
CHR	*3 (6.25%)*	
CCyR	*2 (4.17%)*	
MR3.0	*1 (2.08%)*	
MR4.0	*4 (8.33%)*	
**Sample time after diagnosis, months**	119 ± 63	

Legend: *CHR – Complete Hematologic Response; CCyR – Complete Cytogenetic Response; MR – Molecular Response; SD – standard deviation; NA – Not available*
