# Supplementary material for: NKT-Like (CD3+CD56+) Cells in Chronic Myeloid Leukemia Patients Treated With Tyrosine Kinase Inhibitors
Source: Front Immunol. 2019 Oct 22;10:2493. doi: 10.3389/fimmu.2019.02493 (PMC6817724; doi:10.3389/fimmu.2019.02493)
Supplement: Supplementary file 2 [file Data_Sheet_2.PDF]

S2. Monoclonal antibodies used for flow cytometry studies

Specificity	Fluorochrome	Clone	Isotype	Brand
CD3	V500	UCHT1	IgG_1	BD Horizon
CD11b	V450	ICRF44	IgG_1	BD Horizon
CD16	AF647	3G8	IgG_1	BD Pharmingen
CD27	FITC	O323	IgG_1	Biolegend
CD56	PerCP-Cy5.5	B159	IgG_1	BD Pharmingen
CD57	PB	HNK-1	IgM	Biolegend
CD62L	FITC	DREG-56	IgG_1	Biolegend
CD69	FITC	FN50	IgG_1	Biolegend
CD107a	PE	H4A3	IgG_1	BD Pharmingen
CD137	APC	4B4-1	IgG_1	BD Pharmingen
CD137L	PE	C65-485	IgG_1	BD Pharmingen
CRACC	PE	162.1	IgG_{2b}	Biolegend
Granzyme B	FITC	GB11	IgG_1	BD Pharmingen
HLA-DR	V500	G46-6	IgG_{2a}	BD Horizon
IFN-γ	V450	B27	IgG_1	BD Horizon
LAG-3	PE	T47-530	IgG_1	BD Pharmingen
NKG2A	PE	131411	IgG_{2a}	R&D Systems
NKG2C	APC	134591	IgG_1	R&D Systems
NKG2D	APC	1D11	IgG_1	Biolegend
NKp30	AF647	P30-15	IgG_1	Biolegend
NKp44	AF647	P44-8	IgG_1	Biolegend
NKp46	PE	9E2	IgG_1	Biolegend
NKp80	PE	5D12	IgG_1	Biolegend
PD-1	APC	MIH4	IgG_1	BD Pharmingen
TIM-3	AF647	7D3	IgG_1	BD Pharmingen
Isotype Control	FITC	MOPC-21	IgG_1	Biolegend
Isotype Control	APC	MOPC-21	IgG_1	Biolegend
Isotype Control	PE	MOPC-21	IgG_1	Biolegend
Isotype Control	PB	MOPC-21	IgG_1	Biolegend
Isotype Control	PE	MOPC-173	IgG_{2a}	Biolegend
Isotype Control	PE	MOPC-11	IgG_{2b}	Biolegend
Isotype Control	PB	MM-30	IgM	Biolegend
