# Supplementary material for: NKT-Like (CD3+CD56+) Cells in Chronic Myeloid Leukemia Patients Treated With Tyrosine Kinase Inhibitors
Source: Front Immunol. 2019 Oct 22;10:2493. doi: 10.3389/fimmu.2019.02493 (PMC6817724; doi:10.3389/fimmu.2019.02493)
Supplement: Supplementary file 3 [file Data_Sheet_3.PDF]

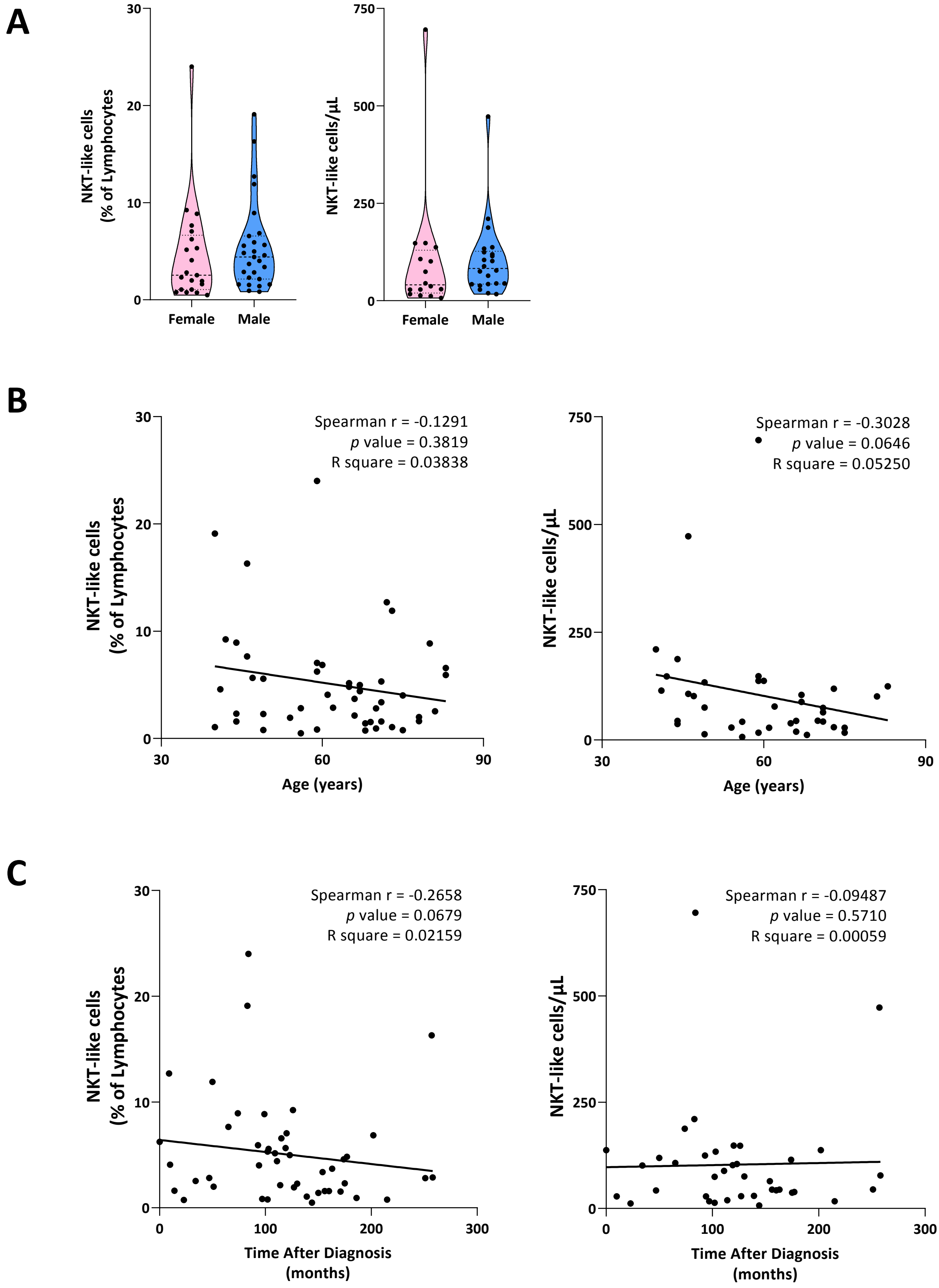
A
NKT-like cells
(% of Lymphocytes
Female
Male
NKT-like cells/µL
B
Spearman r = -0.1291
p value = 0.3819
R square = 0.03838
Age (years)
Spearman r = -0.3028
p value = 0.0646
R square = 0.05250
C
Spearman r = -0.2658
p value = 0.0679
R square = 0.02159
Time After Diagnosis
(months)
Spearman r = -0.09487
p value = 0.5710
R square = 0.00059

S3. Distribution of NKT-like cells from CML patients according to sex, age and Time after diagnosis (TAD). Heparinized fresh whole blood samples were stained with extracellular antibodies and analyzed by multiparametric flow cytometry. **(A)** Relative and absolute frequency of NKT-like cells according to gender (RF – Female: 4,5 ± 5.3, n=21; Male: 5.5 ± 4.7, n=27; AF – Female: 101 ± 166, n=16; Male: 104 ± 98, n=22). No differences were found. **(B)** Correlation between RF (n=48) and AF (n=38) of NKT-like cells with age. No correlation observed. **(C)** Correlation of RF (n=48) and AF (n=38) with sample time after diagnosis in months. Mann Whitney U test (to compare between two groups) and Spearman correlation (to evaluate the association between two parameters) were performed for statistical analysis. *p value <0.05*, <0.01**, <0.001*** or <0.0001****.*
