# Supplementary material for: NKT-Like (CD3+CD56+) Cells in Chronic Myeloid Leukemia Patients Treated With Tyrosine Kinase Inhibitors
Source: Front Immunol. 2019 Oct 22;10:2493. doi: 10.3389/fimmu.2019.02493 (PMC6817724; doi:10.3389/fimmu.2019.02493)
Supplement: Supplementary file 4 [file Data_Sheet_4.PDF]

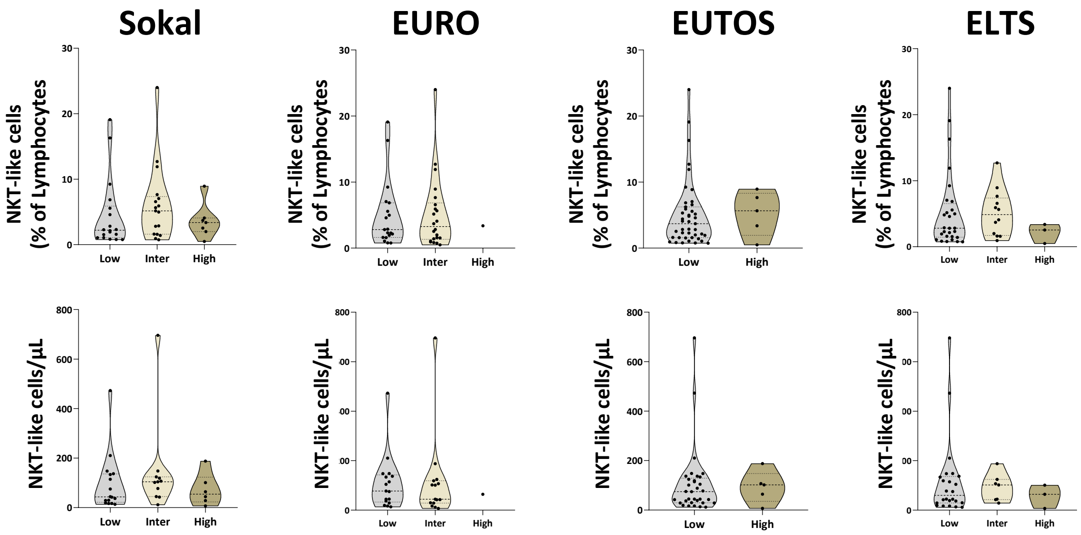


**S4. Distribution of NKT-like cells according to risk scores (Sokal, EURO, EUTOS and ELTS).** Heparinized fresh whole blood samples were stained with extracellular antibodies and analyzed by multiparametric flow cytometry. **Top:** Relative frequency of NKT-like cells according to risk defined for each score. **Down:** Absolute frequency of NKT-like cells according to risk scores. No statistical differences were observed. **Sokal:** Low (n=18), Intermediate (n=17), High (n=7); **EURO:** Low (n=19), Intermediate (n=22), High (n=1); **EUTOS:** Low (n=38), High (n=5); **ELTS:** Low (n=28), Intermediate (n=12), High (n=3).. Mann Whitney U test (to compare between two groups) or one-way ANOVA followed by Dunn’s multiple comparisons test (to compare more than two groups, excluding groups without two or less samples) was achieved for statistical analysis. *Inter – Intermedian; p value <0.05*, <0.01**, <0.001*** or <0.0001****.*
