# Supplementary material for: NKT-Like (CD3+CD56+) Cells in Chronic Myeloid Leukemia Patients Treated With Tyrosine Kinase Inhibitors
Source: Front Immunol. 2019 Oct 22;10:2493. doi: 10.3389/fimmu.2019.02493 (PMC6817724; doi:10.3389/fimmu.2019.02493)
Supplement: Supplementary file 5 [file Data_Sheet_5.PDF]

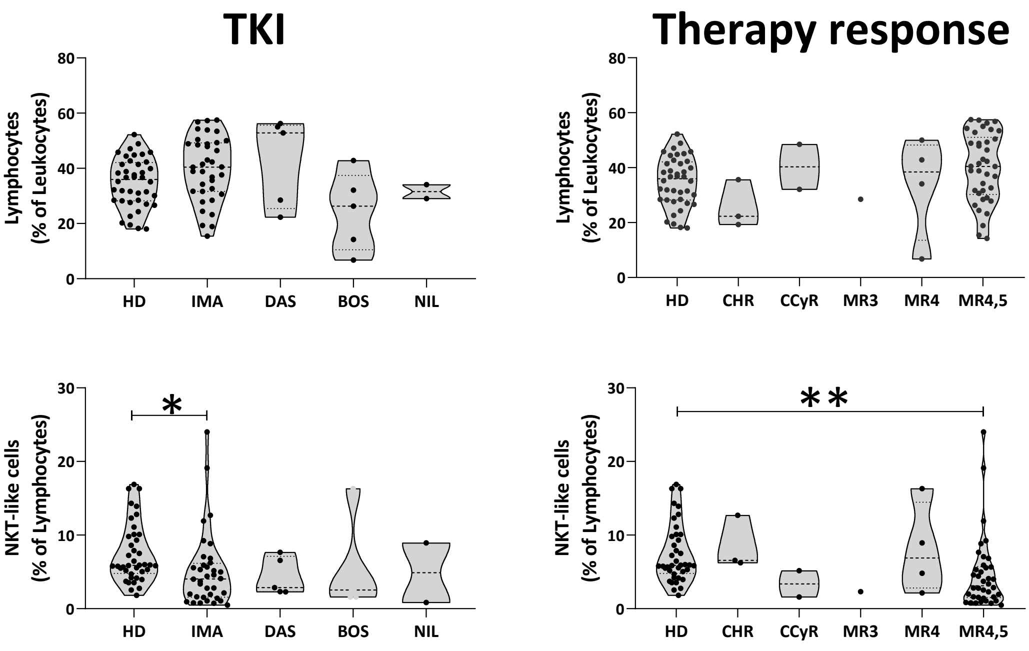

S5. Distribution of lymphocytes and NKT-like cells in CML patients according to individual TKIs and response to therapy. Heparinized fresh whole blood samples were stained with extracellular antibodies and analyzed by multiparametric flow cytometry. **Left:** Relative frequency of lymphocytes and NKT-like cells according to individual TKI **[**HD (n=40); IMA (n=36); DAS (n=5); BOS (n=5); NIL (n=2)**]**. Significantly decrease of NKT-like cells in CML patients undergoing Imatinib therapy. **Right:** Relative frequency of lymphocytes and NKT-like cells according to response to therapy **[**HD (n=40); CHR (n=3); CCyR (n=2); MR3 (n=1); MR4 (n=4); MR4.5 (n=38)**].** Significantly decrease of NKT-like cells in CML patients achieving deep molecular response. One-way ANOVA followed by Dunn's multiple comparisons test was performed to compare more than two groups (excluding groups without 2 or less samples). *HD – Healthy donors; IMA – Imatinib; DAS – Dasatinib; BOS – Bosutinib; NIL – Nilotinib; CHR – Complete Hematological Response; CCyR – Complete Cytogenetic Response; MR – Molecular Response; p value <0.05*, <0.01**, <0.001*** or <0.0001****.*
