# Supplementary material for: NKT-Like (CD3+CD56+) Cells in Chronic Myeloid Leukemia Patients Treated With Tyrosine Kinase Inhibitors
Source: Front Immunol. 2019 Oct 22;10:2493. doi: 10.3389/fimmu.2019.02493 (PMC6817724; doi:10.3389/fimmu.2019.02493)
Supplement: Supplementary file 6 [file Data_Sheet_6.PDF]

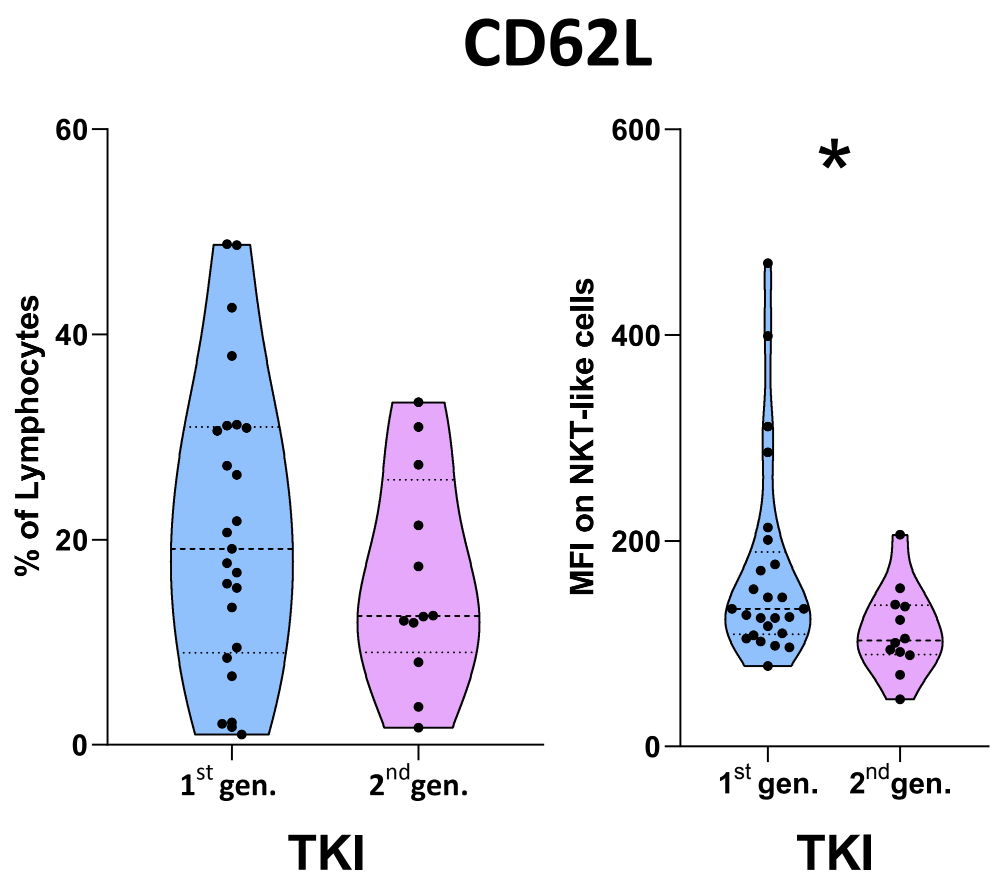

S6. Decreased CD62L membrane expression by NKT-like cells from CML patients under second-generation TKI therapy. Heparinized fresh whole blood samples were stained with extracellular antibodies and analyzed by multiparametric flow cytometry. CD62L relative frequency (left) and MFI (right) in NKT-like cells according to 1st gen. TKI (n=25) and 2nd gen. TKI (n=12). A decrease in CD62L density was observed in 2nd gen TKI comparing to 1st gen. TKI [(MFI: 1st gen (170 ± 98) *vs.* 2nd gen. (112 ± 42), *p*=0,0322]. Mann Whitney U test was used for statistical analysis and the charts represent the mean ± standard deviation. *TKI – Tyrosine Kinase Inhibitor; 1st gen. TKI – 1st generation TKI CML patients; 2nd gen. TKI – 2nd generation TKI CML patients; MFI – Median Intensity Fluorescence; p value <0.05*, <0.01**, <0.001*** or <0.0001****.*
